# Healthcare personnel with laboratory-confirmed mpox in California

**DOI:** 10.1017/ash.2023.313

**Published:** 2023-09-29

**Authors:** Allison Bailey, Jane Siegel, Shua Chai, David Bui, Robert Snyder, Linda Lewis, Kayla Saadeh, J.B. Bertumen, Erin Epson

## Abstract

**Objectives:** Few reports have been published about the transmission of mpox in healthcare settings. During the 2022 multinational outbreak, the California Department of Public Health (CDPH) conducted a systematic review of healthcare personnel (HCP) with mpox, including their community and occupational exposures, to understand the transmission risk in healthcare settings. We also sought to inform return-to-work protocols by describing the frequency of HCP working while symptomatic for mpox and identifying occurrences of secondary transmission from infected HCP to patients. **Methods:** We analyzed surveillance data for laboratory-confirmed mpox cases in California with symptom onset from May 17 to September 30, 2022, collected by investigators at local health departments and reported to the CDPH. The reported data were supplemented by review of free-text variables, interview notes, and other files uploaded to state and county disease surveillance data registries. We identified HCP as all persons working in healthcare settings with potential for direct or indirect exposure to patients or infectious materials, including clinical and nonclinical staff but excluding remote workers. **Results:** The CDPH received reports of 3,176 mpox cases during the study period: 109 were HCP. Of the 109 HCP identified from 19 counties, 78 (72%) were aged 30–49 years, 102 (94%) were male, and 43 (39%) were Hispanic or Latino. Also, 29 HCP (27%) had received at least 1 dose of the JYNNEOS vaccine. Occupations requiring frequent physical interactions with patients were reported for 66 individuals (61%). During interviews with local health department investigators, nearly all HCP (n = 98, 90%) reported potential or confirmed sources of community exposure; 1 had confirmed occupational exposure with symptom onset 9 days after a sharps injury acquired during collection of an mpox specimen for testing. Of the 60 HCP who provided information about the days they worked, 35 (58%) worked while symptomatic, for a mean of 3.14 days (median, 2; IQR, 3). Also, 2 HCP worked for 12 days after symptom onset. No secondary cases of mpox were associated with HCP reported to the CDPH. **Conclusions:** This analysis suggests that HCP are more likely to be exposed to mpox in community settings than healthcare settings. The findings support recommendations against sharps use for mpox specimen collection. Although transmission between symptomatic HCP and patients was not reported, HCP can decrease opportunities for mpox transmission by closely monitoring themselves for symptoms after potential exposures and staying home from work if symptoms develop.

**Disclosures:** None

